# Mastication stimuli regulate the heartbeat rate through rhythmic regulation by the hypothalamic-autonomic system; molecular and telemetric studies in weaning-stage rats

**DOI:** 10.3389/fnins.2023.1260655

**Published:** 2023-09-14

**Authors:** Seonmi Lee, Ryota Tochinai, Akihito Yasuoka, Toshitada Nagai, Yoshikazu Saito, Masayoshi Kuwahara, Keiko Abe, Tomiko Asakura

**Affiliations:** ^1^Department of Applied Biological Chemistry, Graduate School of Agricultural and Life Sciences, The University of Tokyo, Tokyo, Japan; ^2^Department of Veterinary Pathophysiology and Animal Health, Graduate School of Agricultural and Life Sciences, The University of Tokyo, Tokyo, Japan; ^3^Department of Human Nutrition, Seitoku University, Chiba, Japan; ^4^Department of Applied Biological Science, Faculty of Agriculture, Takasaki University of Health and Welfare, Gunma, Japan; ^5^Toyo Institute of Food Technology, Hyogo, Japan; ^6^Project on Health and Anti-Aging, Kanagawa Academy of Science and Technology, Life Science and Environment Research Center (LiSE), Kawasaki, Japan

**Keywords:** mastication, weaning, hypothalamus, transcriptome, heartbeat rate, autonomic nerve

## Abstract

Mastication stimuli have been demonstrated to affect memory function and autonomic nerve activity; however, this process has not been well studied during weaning compared to old age. Previously, we conducted molecular analyses of the thalamus and hippocampus to elucidate the mechanisms underlying this memory-enhancing effect in weaning-stage rats. In this study, we aimed to evaluate the effect of masticatory stimuli on the regulation of heartbeat rate (HR) through the hypothalamic-autonomic system. Three-week-old male rats were administered a powdered diet (P group) or chow-diet (C group) for 10 days. Thereafter, transcriptome analysis was performed. Vasopressin, cocaine-amphetamine-regulated transcript prepropeptide, corticotropin-releasing hormone, and thyrotropin-releasing hormone, which are involved in sympathetic activation of heart rate, were downregulated in the C group. Electrocardiograms were recorded continuously for 12 days under the same condition. Interestingly, rats in the C group had a significantly lower HR than those in the P group on day 11. We checked several parameters representing the autonomic regulation of HR. The C group had higher values for the high-frequency band integration of the HR power spectrum (parasympathetic marker) and root mean square successive difference of R-wave intervals (parasympathetic marker) relative to the P group. Such findings provide a molecular and physiological basis for understanding the regulation of cardiovascular function in response to masticatory stimuli in the autonomic nervous system.

## 1. Introduction

Mastication is a mechanical digestive process that involves jaw and oropharyngeal muscle movements. These movements are regulated by motor nerves with the aid of mechanosensory feedback from the teeth, oropharyngeal epithelium, and muscle sensory organs. These sensory inputs are primarily processed in the medulla oblongata to regulate rhythmic reflex movement of the jaw and are transmitted through tertiary or higher neurons to the thalamus to regulate mastication-triggered physiological responses (Fujita et al., 2017; Yoshida et al., 2022). This sensory information, including both mechanical and chemical stimuli, is well known to play a major role in regulating the digestive system, from the thalamus to the hypothalamic descending pathway (Dhillon et al., 2017; Sato et al., 2019; Nakamura et al., 2022; Doyle et al., 2023). However, several physiological outcomes that are unrelated to the digestive system have been reported.

One of the physiological outcomes that is unrelated to the digestive system is the memory-enhancing effect of mastication stimuli, which is mainly studied in elderly people with reduced mastication motion (Yamamoto et al., 2008; Ono et al., 2010; Chen et al., 2015). Notably, in the weaning stage, animals start to eat a solid diet (Patten et al., 2013; Piancino et al., 2020). Previously, we analyzed the thalamus and hippocampus in weaning-stage rats with special reference to gene expression and neuron micromorphology; this is because the brain at this stage is under the rearrangement of memory function, ultimately preparing for the inevitable nutritional independence, from breastfeeding to autonomous eating. When rats were administered powdered (P group) or chow diet (C group) for 1 week, gamma-aminobutyric acid (GABA) receptor signaling was found to be upregulated and the dendritic spine number in the thalamus decreased in the C group (Ogawa et al., 2018). In a subsequent study, insulin-like growth factor 2 (IGF2)-dependent cellular signaling was upregulated and a larger branch number of apical dendrites was found in the hippocampus of C group rats (Yasuoka et al., 2022). Our findings, in addition to those of other related studies suggest that short-term mastication stimuli in weaning-stage animals are necessary to rearrange the synapses in the thalamus and hippocampus, and are accompanied by an increase in GABA neuron activity in the thalamus, and growth factors, IGF2, and brain-derived nerve growth factor signaling in the hippocampus (Yamamoto et al., 2008; Sunariani et al., 2019).

The other remarkable physiological outcome of mastication stimuli is the effect on heart rate (HR), which might be mediated by the hypothalamic-autonomic (HA) descending pathway. In human studies, HR was demonstrated to increase (HR) within 1 min in response to short-term (< 10 min) chewing gum, and decrease within 1 min after chewing stopped (Hasegawa et al., 2009). The dependence of this response on autonomic regulation can be phenotypically explained by a study on patients with efferent autonomic failure (AF) and *familial dysautonomia* (FD), where a significant reduction in HR was observed in patients with AF, whereas a significantly higher increase in systolic blood pressure in response to chewing gum (SBP) was found in patients with FD (Mora et al., 2015). Humans and animals intermittently masticate food during their awakening time, and the accumulated effects of these stimuli on HA and the subordinate organs and heart have not been well studied in terms of molecular biology and electrophysiology. Here, we performed transcriptome analysis of the hypothalamus and three-week continuous recording of the cardiac potential in weaning-stage rats. Based on our findings, it was suggegsted that rhythmic mastication stimuli were necessary to maintain gene expression related to blood pressure regulation in the hypothalamus and a lower HR during post-weaning animal growth.

## 2. Materials and methods

### 2.1. Animals and breeding method

**Figure 1:**
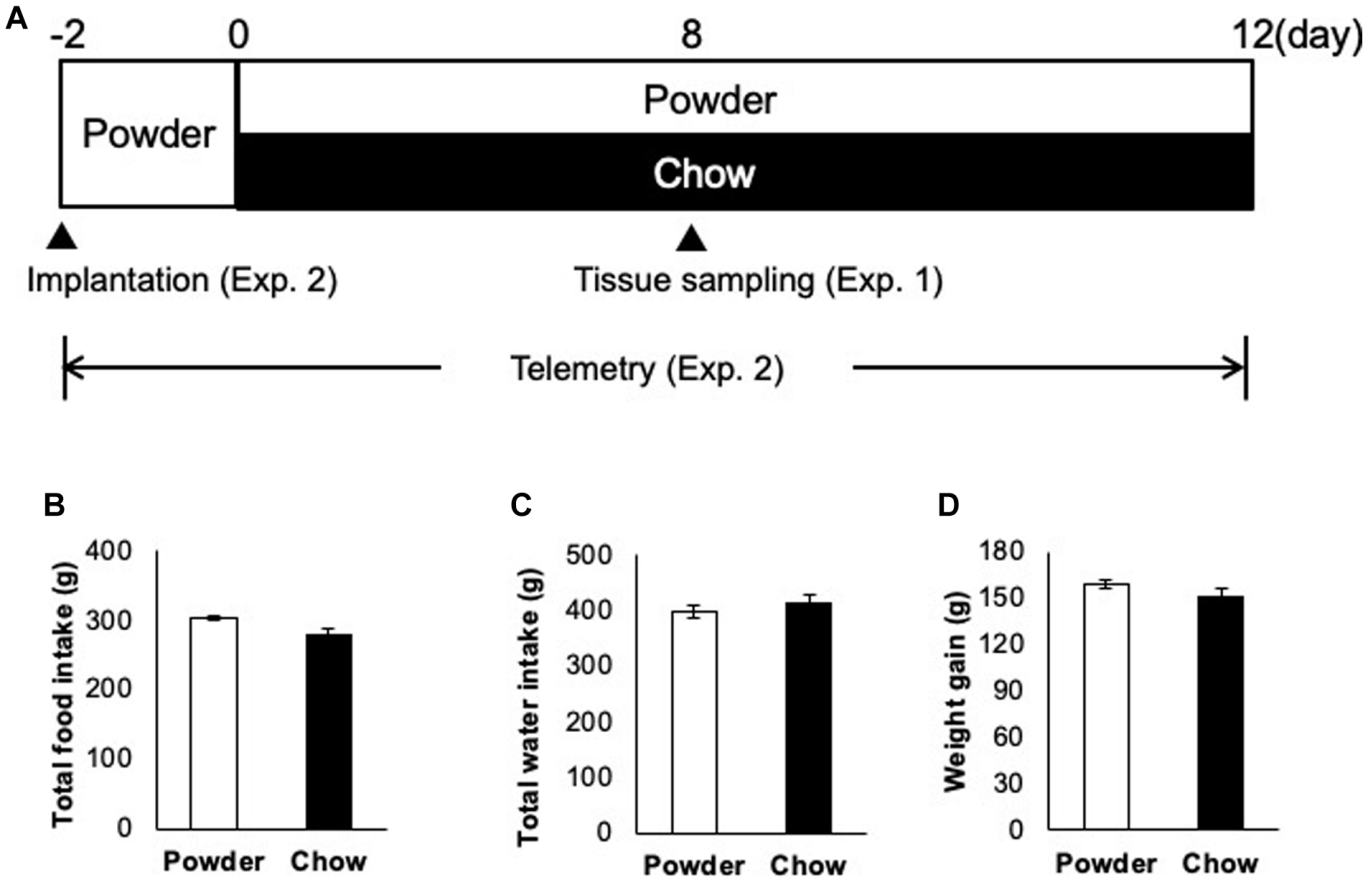
Animal-raising schedule and apparatus. **(A)** Time course of the experiment. In experiment 1 (Exp. 1), 3 week-old male rats (*n* = 5) were acclimatized from days −2 to 0 with a powder diet, raised under different mastication conditions (powder diet and chow diet) from days 0 to 8, and sacrificed on day 8. In experiment 2 (Exp. 2), rats (*n* = 3) were implanted with transmitters on day −2, acclimatized from day −2 to 0, and the heart muscle potential was measured under the same conditions used in Exp. 1 from day 0 to 12. **(B)** Total food intake from days 0 to 20 (time of sacrifice). **(C)** Total water intake from day 0 to 22. **(D)** Total weight gain over 22 days (days 0 to 22).

Three-week-old male Wistar rats (CLEA Japan, Tokyo, Japan) were administered powdered feed and water *ad libitum* for 2 days for acclimatization (Figure 1A). Thereafter, the rats were divided into two groups of equal weight: group one was administered powdered feed (powder, P group) while group two was administered solid feed (chow, C group). These feeds were the same as those used in a previous study (Ogawa et al., 2018). Cages, bedding, and water supplies were designed to avoid chewing, except during dietary consumption. Each cage comprised two standard polycarbonate cages. The beds were fabricated using soft paper chips (EcoChips; CLEA, Japan). Water was administered using a water-absorbent sponge (KS SE10 car wash sponge, Cellulose; Komeri, Niigata, Japan) in a feeding container. A telemetric recording experiment was performed under the same conditions. Two experiments were performed (Figure 1A, Exp. 1 and 2). In Exp. 1, the rats were first fed powder diet for 2 days (acclimatization) and subjected to conditioned feeding for 8 days. In Exp. 2, telemetry transmitters were implanted on day −2, and electrocardiograms were recorded from days −2 to 20. The rats were continuously fed and their body weight and food intake were measured daily until day 20. These experiments were conducted in accordance with the Animal Experimentation Guidelines of the University of Tokyo and were approved by the Institutional Animal Care and Use Committee of the Graduate School of Agricultural and Life Sciences at the University of Tokyo (submission numbers P19–112 for telemetric recording and P17–087 for transcriptome analysis).

### 2.2. Transcriptome analysis

After breeding for 8 days, 10 rats (*n* = 5/groups) were anesthetized with isoflurane (Wako Pure Chemical Industries, Osaka, Japan) during the dissection process. Thereafter, the hypothalamus was extirpated and preserved at −80°C. Total RNA was extracted using TRIzol Reagent (Thermo Fisher Scientific, Waltham, MA, United States) and purified using an RNeasy Mini Kit (Qiagen, Hilden, Germany). Transcriptome analysis was performed as described previously (Ogawa et al., 2018). Briefly, Affymetryx GeneChip Rat Genome 230 2.0 Array (Thermo Fisher Scientific) was used and the obtained CEL file was normalized using the statistical software, R Version 2.7.2, with factor analysis for robust microarray summarization. Hierarchical clustering was performed using the signal values of the normalized probe set. Two groups were compared using the rank products method in R software, and a probe set with a false discovery rate (FDR) < 0.05 was used to identify differentially expressed genes (DEGs). All microarray data were submitted to the Gene Expression Omnibus database of the National Center for Biotechnology Information^1^. Gene ontology (GO) analysis was performed using the Database for Annotation, Visualization and Integrated Discovery (DAVID).^2^ GO terms, where the EASE scores were normalized using multiple comparisons with Benjamini and Hochberg FDR values <0.05, were determined to be significantly enriched.

### 2.3. Telemetry of electrocardiograph and locomotor activity

Electrocardiographic (ECG) body temperature and locomotor activity were recorded in six male Wistar rats (Charles River Laboratories, Kanagawa, Japan). Rats were singly housed in plastic cages in a controlled environment [light–dark cycle 12 h (8:00–20:00)/12 h (20:00–8:00), temperature 24°C] in a thermostatic chamber (MIR-553, Sanyo, Osaka, Japan) with *ad libitum* access to laboratory basal feed and tap water. At 3 weeks-old, immediately after weaning, a small telemetry device (weight = 3.9 g, volume = 1.9 cc; TA10ETA-F20, Data Sciences International, MN, United States) was used to transmit ECG and body temperature. Locomotor activity data were implanted into the dorsal subcutaneous region under systemic anesthesia with isoflurane. The paired wire electrodes of the telemetry device were placed under the skin of the dorsal and ventral thorax to record the apex-base (A-B) lead ECG. Two days after surgery, the animals were divided into the P group and C group (*n* = 3/groups).

ECG signals were recorded from each rat in a cage placed on a signal-receiving board (RA1610, Data Sciences International, MN, United States). ECG data were continuously sampled at 1 ms intervals, and all data analysis of the ECG-wave components was performed using an ECG analysis system (LabChart v8, ADInstruments, New Zealand). During the recording period, body weight, food intake, and water intake were measured daily at 12:00.

### 2.4. Temporal time-course analysis of heart rate variability, locomotor activity, and body temperature

Changes between successive intervals, known as heart rate variability (HRV), can be used as indices of autonomic nervous function. Generally, two main mathematical methods are applied to the analysis of HRV: (A) methods belonging to the time domain and (B) methods belonging to the frequency domain (Kuwahara et al., 1994 and Shaffer and Ginsberg, 2017). In the time domain analysis, the Standard Deviation of the R wave-R wave intervals (SDRR) reflects stimuli from both sympathetic and parasympathetic nervous activity, and the Root Mean Square Successive Difference of R wave-R wave intervals (RMSSD) reflects parasympathetic nervous activity. In the frequency domain analysis, two major spectral components, low frequency (LF:0.1–1.0 Hz) and high frequency (HF:1.0–3.0 Hz), were detected, followed by the LF/HF ratio, which serves as an index of balance between sympathetic and parasympathetic nervous activity in rats. In this study, temporal changes in HRV, locomotor activity, and body temperature were analyzed on days 1, 3, 5, 8, 10, 11, and 12 after conditioned feeding. The average frequency component of the RR interval (LF and HF) based on the Lomb-Scargle Periodogram every 1 min, and the average SDRR and RMSSD every 5 min were calculated using the HRV module (Labchart Pro, ADInstruments, New Zealand). Locomotor activity and body temperature were recorded using the same telemetry implants, and the average value for every 1 min was calculated using an analysis software (Labchart, ADInstruments, New Zealand). The average value of each parameter was calculated for each light and dark period. Data from 12:00 to 15:00 during the light period were excluded from the analysis due to the influence of rearing management operations. The average five-minute SDRR was plotted on the *X*-axis and RMSSD on the *Y*-axis, and the correlation between HRV and parasympathetic nerve activity was analyzed using Pearson’s correlation coefficient.

### 2.5. Statistical analysis

Repeated two-way analysis of variance (ANOVA) was used to examine dependency of the parameters on group, and day factors. Data were also analyzed using Student’s *t*-test. The data are expressed as mean ± SEM. There were extremely high values (> 200) in LF and HF data occupying 0.62% on average. These values seemed to be caused by environmental factors because the distribution of values along time line completely matched between LF and HF data. These values were excluded from the analysis. Especially, the LF and HF data of rat 1 (powder group) day 5 dark period contained many extreme values and no values (14%), therefore all data for day 5 dark period were not used for statistics.

## 3. Results

### 3.1. Effect of mastication stimuli on the hypothalamic transcriptome

**Figure 2:**
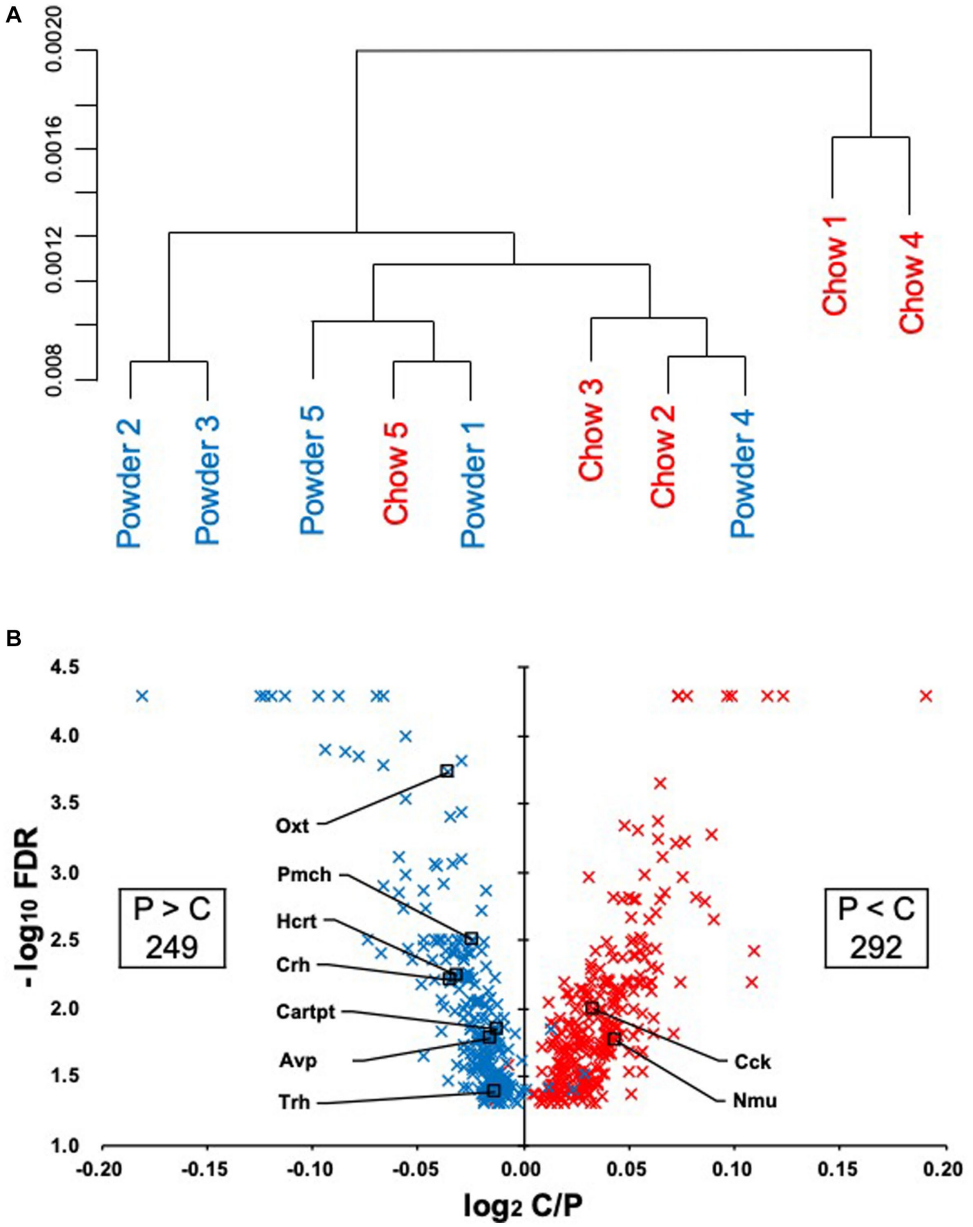
Regulation of the hypothalamic transcriptome by mastication stimuli. **(A)** Five samples from each experimental group (Powder or Chow) were subjected to DNA microarray analysis and represented in a hierarchical cluster. **(B)** The volcano plot of the differentially expressed genes with the number of probe sets showing significant differences in the expression levels between the Powder and Chow groups (FDR < 0.05). Because the genes were extracted by the non-parametric statistics (rank products), some genes were plotted in the opposite quadrant. The FDR values less than 0.0001 were not provided by the program, therefore they were plotted at FDR = 0.00005.

To evaluate the effect of mastication stimuli on the descending pathway from the hypothalamus, 3 week-old male rats were subjected to two feeding conditions: Powder or Chow (P or C) for 8 days (Experiment 1, Figure 1A). Thereafter, DNA microarray analysis of the hypothalamic genes was performed. No differences in food intake (Figure 1B), water intake (Figure 1C), or body weight gain (Figure 1D) were found between the experimental groups, suggesting that the difference in food texture did not cause significant changes in energy intake and consumption as found in our previous study (Yasuoka et al., 2022). The hypothalamic transcriptomes of the individuals were analyzed and represented in a cluster dendrogram (Figure 2A). The overall segregation of transcriptomes between the C and P groups was not clear; two rats in the C group belonged to one independent branch (Chow 1 and 4), and the others were more evenly distributed among the three branches at lower hierarchies (Chow 2, 3, and 5, and Powder 1 to 5). Nevertheless, the statistical test (FDR < 0.05) revealed 292 (Powder < Chow) and 249 (Powder > Chow) DEGs (Figure 2B). As a result, we identified nine genes related to heart rate regulation (Table 1 and Figure 2B), two and seven of which were upregulated and downregulated, respectively, in the C group. All genes encode hormones that regulate the heart rate through the autonomic system and peripheral organs. The regulatory effect of the genes on heartbeat rate could be predicted based on their gene expression levels in the experimental groups; six genes (*Cck*, *Avp*, *Cartpt*, *Crh*, *Hcrt*, and *Trh*) were downregulated, and three genes (*Nmu*, *Oxt*, and *Pmch*) were upregulated (Table 1). Functional enrichment analysis of DEGs was performed using Gene Ontology (GO) terms; however, GO terms related to the hypothalamic regulation of autonomic nerves were not found, except GO:0051384, Response to glucocorticoids with Oxt (Supplementary Table S1), which included the oxytocin gene (Table 1). Altogether, differences in mastication stimuli can affect autonomic nerve regulation by the hypothalamus without causing significant changes in the physical parameters.

**Table 1:** Hypothalamic DEGs related to autonomic nerve regulation.

Gene symbol	Gene name	Expression	Function	Heart beat rate regulation in C-group
Cck	Cholecystokinin	*P* < C	Parasympathetic	Down
Nmu	Neuromedin U		Sympathetic	Up
Avp	Arginine vasopressin	*P* > C	Sympathetic, peripheral	Down
Cartpt	Cocaine-amphetamine regulated transcript prepropeptide		Sympathetic	Down
Crh	Corticotropin-releasing hormone		Sympathetic, peripheral	Down
Hcrt	Hypocretin neuropeptide precursor		Sympathetic	Down
Oxt	Oxytocin/neurophysin 1 prepropeptide		Parasympathetic, peripheral	Up
Pmch	Pro-melanin-concentrating hormone		Parasympathetic	Up
Trh	Thyrotropin releasing hormone		Sympathetic	Down

### 3.2. Effect of mastication stimuli on the physiological parameters related to autonomic nerve regulation

**Figure 3:**
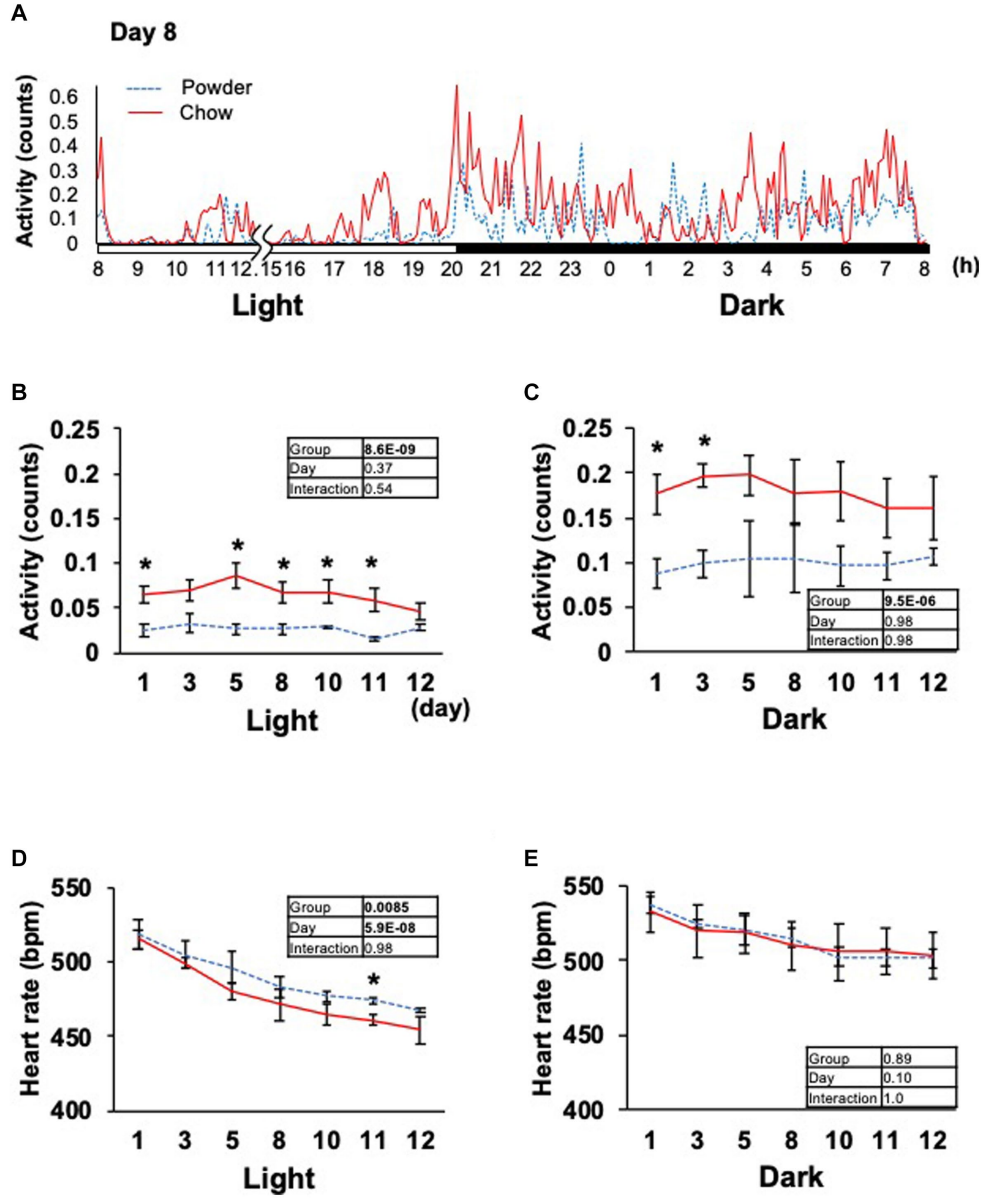
Time course of animal activity and heartbeat rate. **(A)** In-day traces of animal activity within 5 min on day 8. Gaps indicate disturbances in data acquisition due to animal maintenance between hours 12 and 15 (12.15). **(B,C)** Average animal activity during the light or dark period from days 1 to 12. **(D,E)** Averages of the heartbeat rate. Inset tables; The results of repeated two-way ANOVA with significant *p*-values in boldface (*p* < 0.05). *Significant difference was detected using the *t*-test (*p* < 0.05).

**Figure 4:**
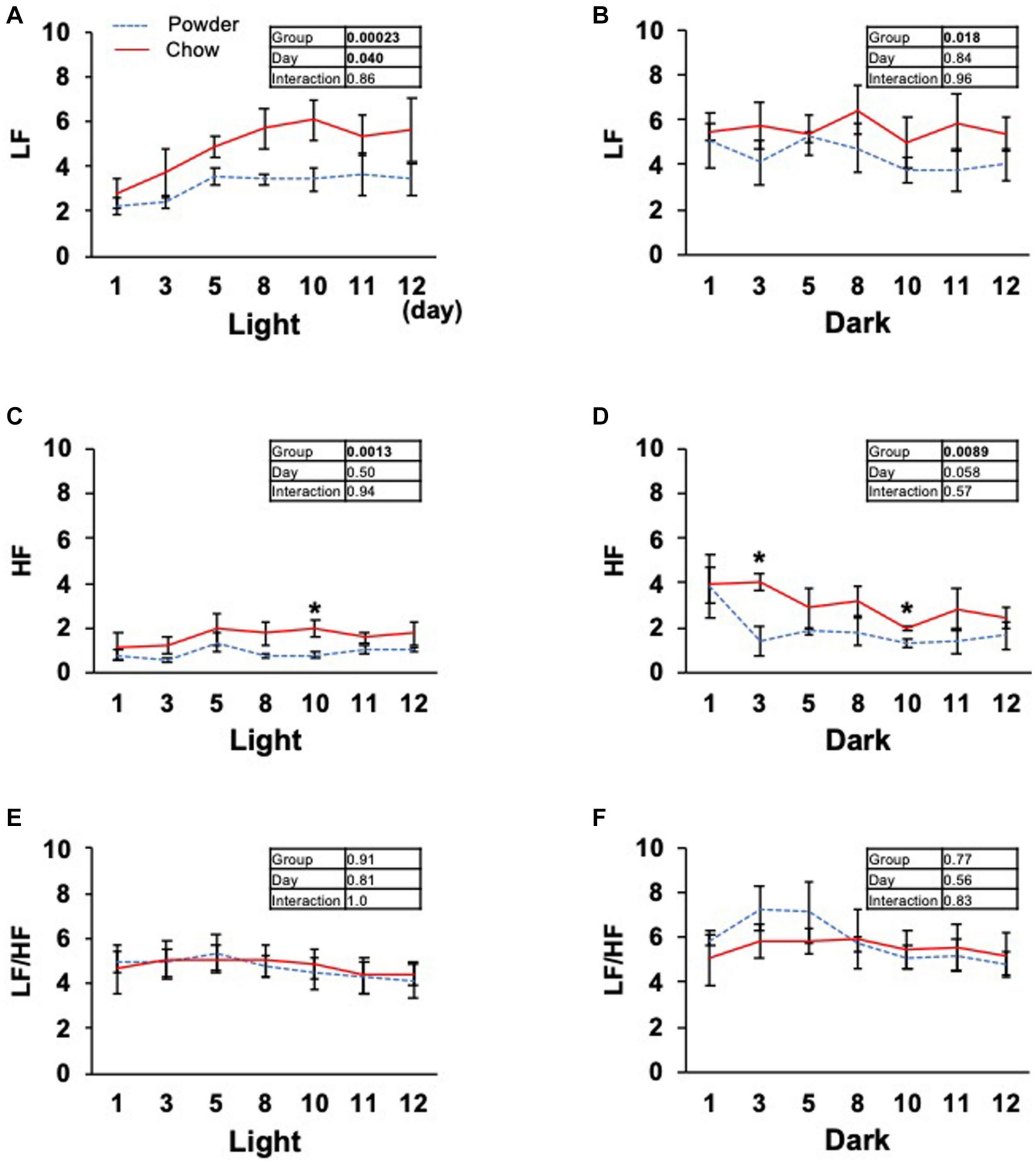
Time course of low frequency (LF), high frequency (HF) band integration, and LF/HF ratio of the heart rate power spectrum. **(A–F)** Average light and dark period data from day 1 to 12. Inset tables; The results of repeated two-way ANOVA with significant p-values in boldface (*p* < 0.05). *Significant difference was detected using the *t*-test (*p* < 0.05).

**Figure 5:**
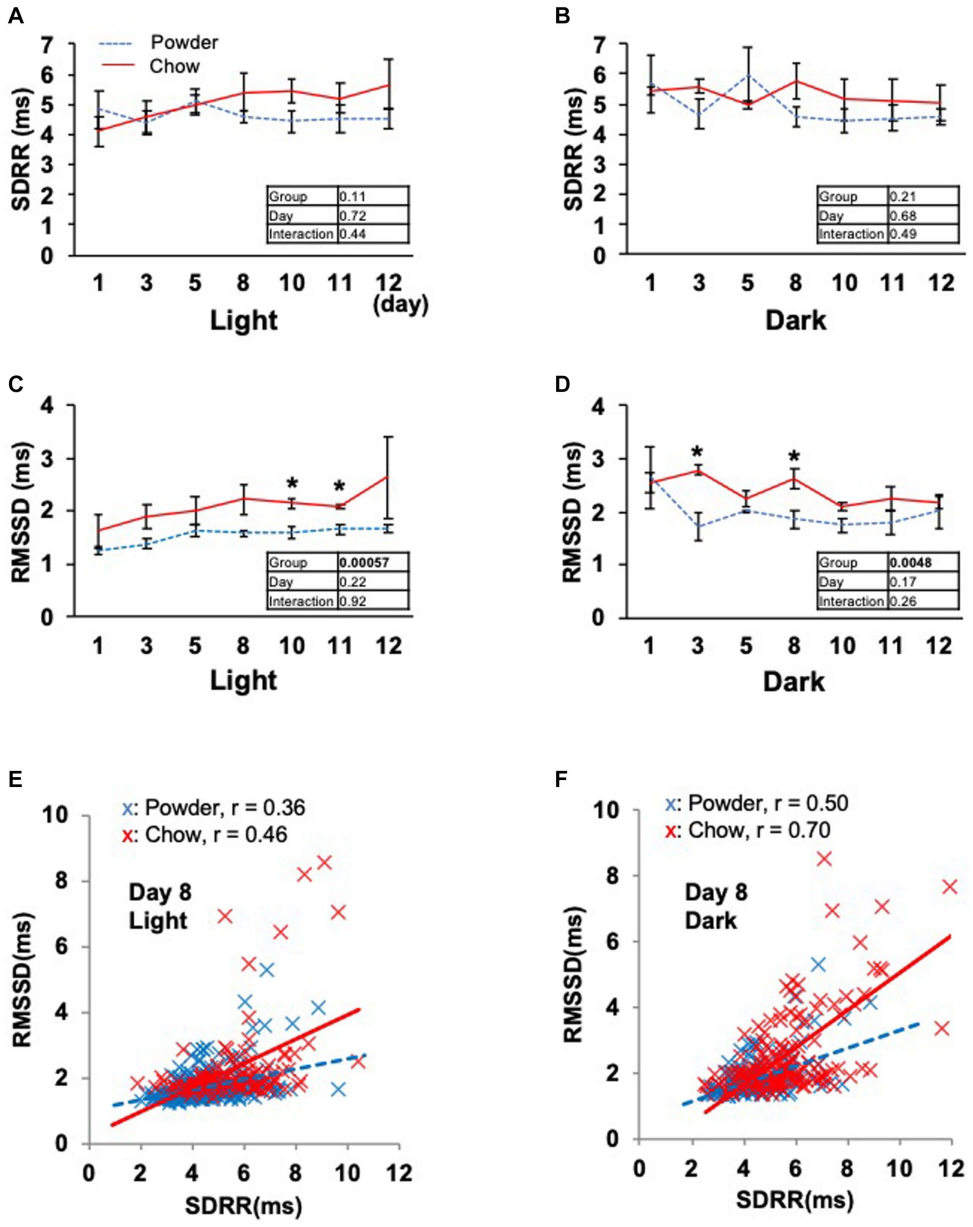
Time course of the heartbeat rate fluctuation indexes. **(A,B)** Averages of the standard deviations of R wave-R wave intervals within 5 min (SDRR) from day 1 to 12. **(C,D)** Averages of the standard deviation of the root mean square successive difference of R wave-R wave intervals within 5 min (RMSSD). Inset tables; The results of repeated two-way ANOVA with significant *p*-values in boldface (*p* < 0.05). *Significant difference was detected using the *t*-test (*p* < 0.05). **(E,F)** Correlation between the SDRR and RMSSD values on day 8 of the light and dark periods. Each dot represents the values obtained every 5 min from six rats. The solid and dotted lines represent linear approximations of the C and P groups, respectively. The Pearson’s correlation coefficient (r) was calculated for each experimental group.

Based on the significant changes in hypothalamic genes, we aimed to determine the physiological outcomes of autonomic nerve regulation by mastication stimuli. Accordingly, we subcutaneously implanted the heart muscle potential probe into rats during the acclimatization period. The animals were maintained under the same condition used in the transcriptome analysis, and the electrocardiogram was measured for 22 days (Exp. 2, Figure 1A). Because the stability of recording began to decrease after day 15, only the data from day 0 to 12 were analyzed. We measured rat movement (activity) by calculating the rate of change in the radio wave strength. In the one-day trace of activity on day 8, major peaks were observed at 20–23 h and 3–8 h (Figure 3A). When the values were averaged during the light and dark periods, the C group showed significantly higher activity values than the P group (Figures 3B,C). For HR, a significantly lower average value was found for the C group during the light period on day 11 (Figure 3D). The repeated two-way ANOVA of HR data detected the dependency of HR values not only on the group factor but also on the day factor (Figure 3D inset table). Because both C and P group exhibited decrease of HR through the experimental period, these may have arisen from the development of heart function during the post-weaning maturation. These results indicate that masticatory stimuli can alter animal HR, possibly via autonomic nerve regulation. We examined several parameters related to the mode of HR regulation that can be interpreted as sympathetic and/or parasympathetic activity. The low-frequency (LF) and high-frequency (HF) band integration of the heartbeat rate power spectrum and their ratio (LF/HF) are conventional tools for assessing autonomic regulation of HR. A higher LF represents autonomic activation and a higher HF represents parasympathetic dominance of heartbeat regulation (Kuwahara et al., 1994). We detected significant dependencies of LF values on the group factors (Figures 4A,B), and significantly higher average HF values on day 3 dark period, and on day 10 (Figures 4C,D); however, no significant difference was found for LF/HF (Figures 4E,F). The SDRR within 5 min and RMSSD within 5 min are parameters that reflect the fluctuation of heartbeat regulation in the time domain. SDRR has been demonstrated to reflect total autonomic nerve activation, and RMSSD is useful for detecting the dominance of parasympathetic nerve (Shaffer and Ginsberg, 2017). Interestingly, the average SDRR values did not show any between-group differences (Figures 5A,B), while the average RMSSD values of the C group were significantly higher than those of the P group during the light and dark periods (Figures 5C,D). As these parameters represent the disturbance of heart rate periodicity, we tested the linear independence between SDRR and RMSSD by plotting each value obtained every 5 min from six rats. A relatively high correlation coefficient (*r* = 0.70) was observed in the dark period (Figures 5E,F). Such finding indicates that the fluctuation of the heart rate in the time domain and parasympathetic nerve dominance occurred more synchronously in group C during the dark period.

## 4. Discussion

In this study, we elucidated the molecular response of the HA system to mastication stimuli and its physiological outcomes in terms of heart rate regulation in weaning-stage rats. Physical parameters, such as food intake and body weight gain, did not differ between young rats in the experimental groups (Figure 1). Therefore, the difference in diet texture between the powder and chow diet did not cause significant changes in the overall energy income or outgoing of rats. However, transcriptome analysis of the hypothalamus revealed changes in the expression of multiple genes related to heat rate regulation in the HA system (Table 1). As the regulation of hypothalamic genes at the time of sacrifice was partially contradictory (three genes for HR upregulation and six for downregulation), we hypothesized that the physiological phenotype of the rats should be evaluated continuously throughout the experimental period. Accordingly, we measured the cardiac potential for 12 days using a hypodermically implanted probe. This probe also enabled us to monitor animal movement (activity) by differentiating the time course of the radio wave strength. The major peaks of activity were observed at 20–23 h and 3–8 h in the one-day trace of day 8 (Figure 3A), which overlapped with the time distribution of the animals’ feeding behavior reported previously (Ritskes and Strubbe, 2007 and Tilston et al., 2019). The higher activity observed in C group might be associated with the feeding behavior related to the chow diet, for example, the time spent handling and masticating the diet.

**Figure 6:**
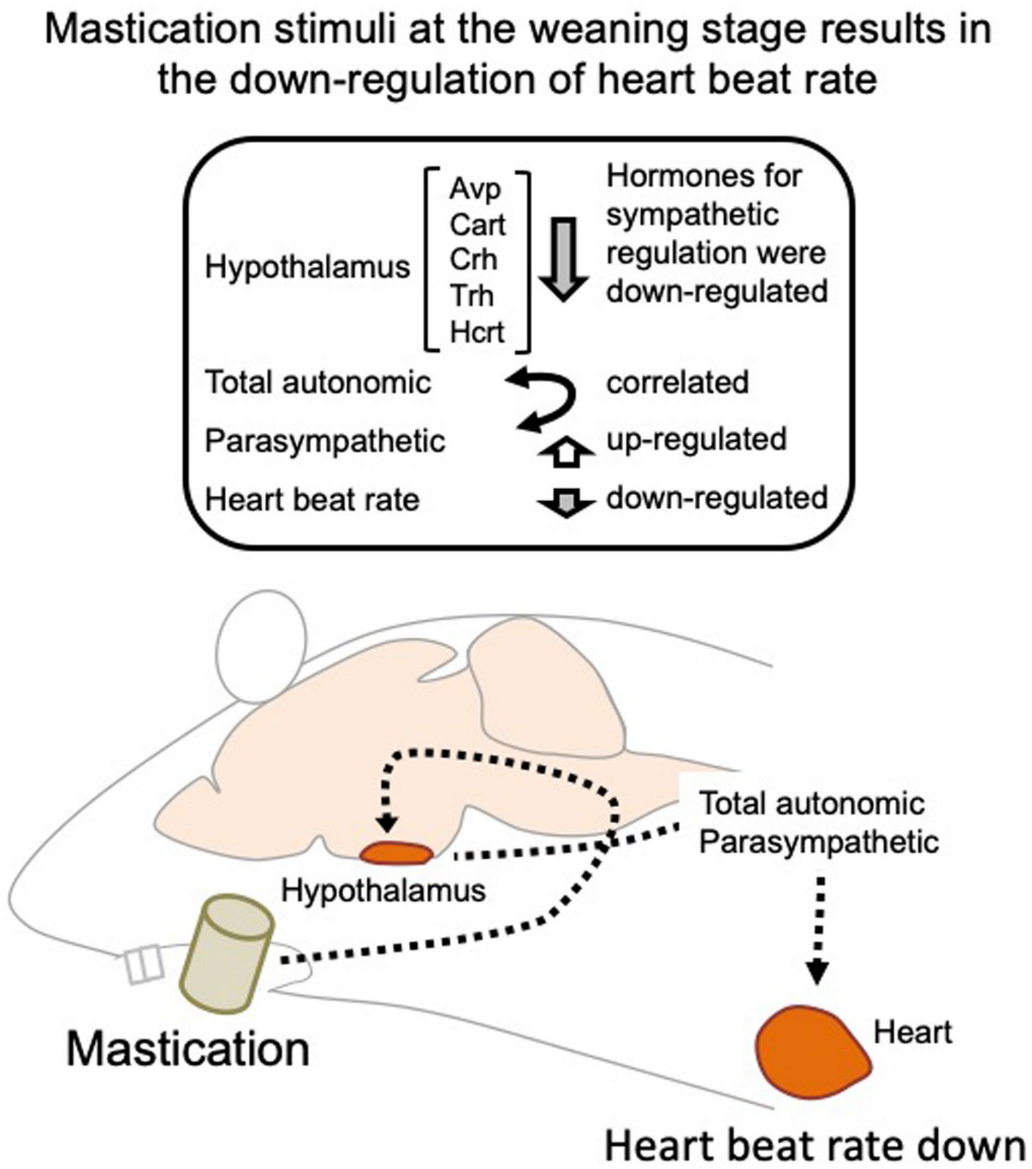
Regulation of the heartbeat rate by mastication stimuli during the weaning period. Intermittent mastication stimuli activate both total autonomic and parasympathetic activation through the hypothalamus, resulting in a decreased heart rate at 5 week-old. Avp, arginine vasopressin; Cart, cocaine-amphetamine-regulated transcript pre-propeptide; Crh, corticotropin-releasing hormone; Trh, thyrotropin-releasing hormone; Hcrt, hypocretin neuropeptide precursor.

Several human studies have been conducted on the autonomic nerve parameters of the heartbeat during mastication, in which an increase in HR, decrease in HF, and increase in LF/HF were reported (Hasegawa et al., 2009; Niizeki and Saitoh, 2016; Ohta et al., 2018). These results suggest sympathetic nerve dominance during short-term mastication. Hasegawa et al. reported that 10 min of chewing gum stimulation upregulated HR within 1 min; however, this increase disappeared after stimuli termination (Hasegawa et al., 2007). In the present study, the average HR significantly decreased on day 11 in the C group (Figure 3D), suggesting the overall dominance of the parasympathetic nerve. Concordantly, higher HF (parasympathetic marker, Figure 4D) and RMSSD (parasympathetic marker, Figures 5C,D) were observed in the C group. The apparent discrepancy between previous human studies (timescale of less than 30 min) and our study (timescale longer than 10 days) may be uniformly interpreted by the correlation analysis between RMSSD representing parasympathetic activation and SDRR representing total autonomic activation. A higher Pearson’s correlation coefficient (*r* = 0.70) was found between RMSSD and SDRR in the C group on day 8 of the dark period (Figure 5F). As the values were calculated from the data averaged every 5 min (5 values/5 min), the coincidence of parasympathetic activation and total autonomic activation may have occurred at a time scale close to 5 min. Accordingly, more frequent upregulation of HR by mastication and its downregulation by the cessation of mastication may have occurred in rats fed intermittently at approximately 20-min intervals (Tilston et al., 2019). Accordingly, the significantly lower HR in the C group (Figure 3D) could be due to the overall dominance of the parasympathetic system. Notably, a lower HR was only observed during the light period, resting, and feeding termination (Figure 3D). To assess the mechanism underlying this cyclic regulation, further biochemical analyses of serum hormone levels, such as corticosterone, corticotropin-releasing hormone (*Crh* product), thyroid hormone (*Trh* product), oxytocin (*Oxt* product), and arginine vasopressin (*Avp* product), should be performed. In conclusion, the decreased HR on day 11 in the C group may have occurred in the following sequence: starting a chow diet on day 1, physiological changes from day 1 to day 8, gene expression changes from day 1 to day 8, and stabilization of the physiological phenotype on day 11 (Figure 6).

In this study, we identified nine DEGs related to blood pressure regulation in the hypothalamus (Table 1). Five of these DEGs, namely *Nmu*, *Avp*, *Crh*, *Oxt*, and *Trh*, are known to be expressed in the paraventricular nucleus and play a pivotal role in the stress responses of the hypothalamic–pituitary–adrenal system. Chewing gum is known to relieve social aggression and occasional circumstantial stresses (Ekuni et al., 2012; Allen and Smith, 2015; Walker et al., 2016). The neuronal pathway responsible for this effect remains to be elucidated; however, this pathway may share some similarities with the heartbeat regulation by mastication observed in our studies. As our previous study revealed GABA signaling activation by mastication stimuli in the thalamus (Ogawa et al., 2018), examining the GABA neuronal activity in the thalamus and its connectivity with the hypothalamus using our model system could be a valuable assessment. Overall, our findings suggest the importance of mastication in the normal development of HA-dependent regulation of the heart during the post-weaning stage.

## Data availability statement

The datasets presented in this study can be found in online repositories. The names of the repository/repositories and accession number(s) can be found at: https://www.ncbi.nlm.nih.gov/, GSE236446.

## Ethics statement

The animal study was approved by the Institutional Animal Care and Use Committee of the Graduate School of Agricultural and Life Sciences at the University of Tokyo (submission numbers P19-112 for telemetric recording and P17-087 for transcriptome analysis). The study was conducted in accordance with the local legislation and institutional requirements.

## Author contributions

SL: Data curation, Investigation, Writing – original draft. RT: Data curation, Formal Analysis, Investigation, Methodology, Writing–review & editing. AY: Conceptualization, Data curation, Formal Analysis, Investigation, Supervision, Validation, Writing – original draft, Writing–review & editing. TN: Data curation, Formal Analysis, Funding acquisition, Supervision, Writing–review & editing. YS: Data curation, Formal Analysis, Investigation, Methodology, Writing–review & editing. MK: Project administration, Supervision, Validation, Writing–review & editing. KA: Project administration, Supervision, Validation, Writing–review & editing. TA: Conceptualization, Formal Analysis, Funding acquisition, Project administration, Supervision, Validation, Writing–review & editing.

## Funding

The author(s) declare financial support was received for the research, authorship, and/or publication of this article. This study was supported by the JSPS KAKENHI; Grant Numbers 18H00961 (TA) and 21 K11707 (TN).

## Conflict of interest

The authors declare that the research was conducted in the absence of any commercial or financial relationships that could be construed as a potential conflict of interest.

## Publisher’s note

All claims expressed in this article are solely those of the authors and do not necessarily represent those of their affiliated organizations, or those of the publisher, the editors and the reviewers. Any product that may be evaluated in this article, or claim that may be made by its manufacturer, is not guaranteed or endorsed by the publisher.

## Footnotes

^1^NCBI, http://www.ncbi.nlm.nih.gov/geo/; GEO Series ID GSE236446.

^2^ https://david.ncifcrf.gov/
