# Supplementary material for: Mastication stimuli regulate the heartbeat rate through rhythmic regulation by the hypothalamic-autonomic system; molecular and telemetric studies in weaning-stage rats
Source: Front Neurosci. 2023 Sep 14;17:1260655. doi: 10.3389/fnins.2023.1260655 (PMC10536135; doi:10.3389/fnins.2023.1260655)
Supplement: Supplementary file 1 [file Table_1.docx]

Supplementary Table 1 Enrichment of the DEGs to GO terms

| GO:0030199 collagen fibril organization | Col1a2 /// NEWGENE_621351 | collagen, type I, alpha 2 /// collagen, type I, alpha 2 |
| --- | --- | --- |
|  | Anxa2 | annexin A2 |
|  | NEWGENE_621351 | collagen, type I, alpha 2 |
|  | Lum | lumican |
|  | Col3a1 | collagen, type III, alpha 1 |
|  | Col5a1 | collagen, type V, alpha 1 |
|  | Fmod | fibromodulin |
|  | Col1a1 | collagen, type I, alpha 1 |
|  | Col1a1 | collagen, type I, alpha 1 |
|  | Ddr2 | discoidin domain receptor tyrosine kinase 2 |
|  | Serpinh1 | serpin peptidase inhibitor, clade H (heat shock protein 47), member 1, (collagen binding protein 1) |
| GO:0032964 collagen biosynthetic process | Col5a1 | collagen, type V, alpha 1 |
|  | Col1a1 | collagen, type I, alpha 1 |
|  | Col1a1 | collagen, type I, alpha 1 |
|  | Serpinh1 | serpin peptidase inhibitor, clade H (heat shock protein 47), member 1, (collagen binding protein 1) |
|  | Arg1 | arginase 1 |
| GO:0045104 intermediate filament cytoskeleton organization | Synm | synemin, intermediate filament protein |
|  | Nefh | neurofilament, heavy polypeptide |
|  | Prph | peripherin |
|  | Nefm | neurofilament, medium polypeptide |
|  | Ina | internexin neuronal intermediate filament protein, alpha |
| GO:0002474 antigen processing and presentation of peptide antigen via MHC class I | RT1-T24-4 | RT1 class I, locus T24, gene 4 |
|  | RT1-A2 /// RT1-A3 /// RT1-EC2 | RT1 class Ia, locus A2 /// RT1 class I, locus A3 /// RT1 class Ib, locus EC2 |
|  | RT1-T24-3 | RT1 class I, locus T24, gene 3 |
|  | RT1-S3 | RT1 class Ib, locus S3 |
|  | RT1-S3 | RT1 class Ib, locus S3 |
|  | RT1-S3 | RT1 class Ib, locus S3 |
|  | RT1-CE16 | RT1 class I, locus CE16 |
|  | RT1-EC2 | RT1 class Ib, locus EC2 |
|  | RT1-S3 | RT1 class Ib, locus S3 |
| GO:0051384 response to glucocorticoid | Ptgds | prostaglandin D2 synthase (brain) |
|  | Lcat | lecithin cholesterol acyltransferase |
|  | Sult1a1 | sulfotransferase family 1A member 1 |
|  | Bmp6 | bone morphogenetic protein 6 |
|  | Mgp | matrix Gla protein |
|  | Bmp4 | bone morphogenetic protein 4 |
|  | Bmp6 | bone morphogenetic protein 6 |
|  | Aqp4 | aquaporin 4 |
|  | Igfbp2 | insulin-like growth factor binding protein 2 |
|  | A2m /// LOC100911545 | alpha-2-macroglobulin /// alpha-2-macroglobulin-like |
|  | Anxa1 | annexin A1 |
|  | C3 | complement component 3 |
|  | Oxt | oxytocin/neurophysin 1 prepropeptide |
|  | Got1 | glutamic-oxaloacetic transaminase 1, soluble |
| GO:0001568 blood vessel development | Col1a2 /// NEWGENE_621351 | collagen, type I, alpha 2 /// collagen, type I, alpha 2 |
|  | Mef2c | myocyte enhancer factor 2C |
|  | NEWGENE_621351 | collagen, type I, alpha 2 |
|  | Itgav | integrin, alpha V |
|  | Aldh1a2 | aldehyde dehydrogenase 1 family, member A2 |
|  | Stra6 | stimulated by retinoic acid 6 |
|  | Stra6 | stimulated by retinoic acid 6 |
|  | Col3a1 | collagen, type III, alpha 1 |
|  | Col5a1 | collagen, type V, alpha 1 |
|  | Col1a1 | collagen, type I, alpha 1 |
|  | Col1a1 | collagen, type I, alpha 1 |
|  | Bmp4 | bone morphogenetic protein 4 |
| GO:0009612 response to mechanical stimulus | Angpt2 | angiopoietin 2 |
|  | Col3a1 | collagen, type III, alpha 1 |
|  | Cxcl12 | chemokine (C-X-C motif) ligand 12 |
|  | Tnc | tenascin C |
|  | Col1a1 | collagen, type I, alpha 1 |
|  | Dcn | decorin |
|  | Txnip | thioredoxin interacting protein |
|  | Mgp | matrix Gla protein |
|  | Bmp4 | bone morphogenetic protein 4 |
|  | Igfbp2 | insulin-like growth factor binding protein 2 |
|  | Col1a1 | collagen, type I, alpha 1 |
|  | Meis2 | Meis homeobox 2 |
|  | Oxt | oxytocin/neurophysin 1 prepropeptide |
| GO:0042573 retinoic acid metabolic process | Rbp1 | retinol binding protein 1, cellular |
|  | Aldh1a2 | aldehyde dehydrogenase 1 family, member A2 |
|  | Stra6 | stimulated by retinoic acid 6 |
|  | Aldh1a1 | aldehyde dehydrogenase 1 family, member A1 |
|  | Stra6 | stimulated by retinoic acid 6 |
|  | Cyp26b1 | cytochrome P450, family 26, subfamily b, polypeptide 1 |
| GO:0001958 endochondral ossification | Mef2c | myocyte enhancer factor 2C |
|  | Pthlh | parathyroid hormone-like hormone |
|  | Bmp6 | bone morphogenetic protein 6 |
|  | Col1a1 | collagen, type I, alpha 1 |
|  | Col1a1 | collagen, type I, alpha 1 |
|  | Bmp6 | bone morphogenetic protein 6 |
|  | Bmp4 | bone morphogenetic protein 4 |
|  | Cbs | cystathionine beta synthase |
|  | Gnas | GNAS complex locus |
| GO:0015671 oxygen transport | Hbb-b1 /// LOC100134871 /// LOC103694857 /// LOC689064 | hemoglobin, beta adult major chain /// beta globin minor gene /// hemoglobin subunit beta-2 /// beta-globin |
|  | Hba1 /// Hba2 | hemoglobin, alpha 1 /// hemoglobin, alpha 2 |
|  | Hba1 /// Hba2 | hemoglobin, alpha 1 /// hemoglobin, alpha 2 |
|  | Hbb | hemoglobin, beta |
|  | Hbb-b1 /// LOC103694857 /// LOC689064 | hemoglobin, beta adult major chain /// hemoglobin subunit beta-2 /// beta-globin |
|  | Hba1 /// Hba2 | hemoglobin, alpha 1 /// hemoglobin, alpha 2 |
|  | Hba-a1 | hemoglobin alpha, adult chain 1 |
|  | Ngb | neuroglobin |

541 differentially expressed genes were analyzed for their enrichment in GO-terms using DAVID (*p*-value < 0.1, https://david.ncifcrf.gov/). Genes in shaded and plane columns were upregulated and downregulated in C-group, respectively.
